# Effect of the *OPHN1* novel variant c.1025+1 G>A on RNA splicing: insights from a minigene assay

**DOI:** 10.1186/s12920-024-01952-1

**Published:** 2024-07-02

**Authors:** Fei Yang, Minghui Wang

**Affiliations:** Changde Hospital, Xiangya School of Medicine, Central South University(The First People’s Hospital of Changde City), No.818 Renmin Road, Changde, Hunan 415000 China

**Keywords:** *OPHN1* gene, X-linked intellectual disability, Whole-exome sequencing, Minigene

## Abstract

This research analyzes the clinical data, whole-exome sequencing results, and in vitro minigene functional experiments of a child with developmental delay and intellectual disability. The male patient, aged 4, began experiencing epileptic seizures at 3 months post-birth and has shown developmental delay. Rehabilitation training was administered between the ages of one and two. There were no other significant family medical histories. Through comprehensive family exome genetic testing, a hemizygous variant in the 11th exon of the *OPHN1* gene was identified in the affected child: c.1025 + 1G > A. Family segregation analysis confirmed the presence of this variant in the patient’s mother, which had not been previously reported. According to the ACMG guidelines, this variant was classified as a likely pathogenic variant. In response to this variant, an in vitro minigene functional experiment was designed and conducted, confirming that the mutation affects the normal splicing of the gene’s mRNA, resulting in a 56 bp retention on the left side of Intron 11. It was confirmed that *OPHN1*: c.1025 + 1G > A is the pathogenic cause of X-linked intellectual disabilities in the child, with clinical phenotypes including developmental delay and seizures.

**Supplementary Information:**

The online version contains supplementary material available at 10.1186/s12920-024-01952-1.

## Introduction

Intellectual disability is a common disabling condition in children, referring to significant impairments in intellectual functioning and adaptive behaviour compared to same-age peers during the developmental process (before 18 years old) caused by various factors [1]. The etiology of intellectual disability is complex and associated with both environmental and genetic factors. Many instances of intellectual disability associated with genetic factors manifest as X-linked intellectual disabilities (XLID) [2–4], XLID denotes congenital intellectual deficits resulting from pathogenic mutations in genes situated on the X chromosome, contributing to 10% of intellectual disabilities or developmental delays in male children [5]. Studies suggest that between 10% and 15% of intellectual disabilities are linked to genetic variants on the X chromosome, which represents about 4% of the human genome [6, 7]. Research has also found that the expression level of human X chromosome genes in the central nervous system is 2.8 times that in other tissues, further indicating the significant role of X chromosome genes in normal brain development and function [8].

Currently, over 100 genes related to XLID have been reported, and the diagnosis of non-syndromic XLID poses challenges due to its complex clinical features [9, 10]. The *OPHN1* gene, located on Xq12, consists of 25 exons and encodes Rho GTPase-activating protein (RhoGAP), which is composed of 802 amino acids. *OPHN1* gene is highly expressed in the fetal and adult brain, and RhoGAP can promote GTP hydrolysis and regulate the activity of Rho proteins [11]. Mutations in the *OPHN1* gene can lead to protein dysfunction [12]. Multiple post-transcriptional events involving the OPHN1 protein, along with editing/splicing of mutant OPHN1 protein, may play a significant role in the pathogenesis of intellectual disability [13].

This study investigates a 4-year-old boy who experienced seizures and loss of consciousness three months after birth, exhibiting fixed gaze and subsequently admitted to the pediatric intensive care unit. The patient exhibited signs of developmental delay and neurological impairment, including unresponsiveness to his name, lack of eye contact, seizures, poor motor coordination, and fine motor skills. While he was capable of grasping objects with both hands, he faced challenges with crawling, sitting independently, and standing with support. The study employed whole-exome sequencing (WES) to identify genetic variations in the patient for genetic diagnosis. Regarding the variant site, a mutant vector was constructed in vitro to assess its impact on protein function.

## Materials and methods

### Patient clinical information

The patient is a 4-year-old boy, born at full term with a birth weight of 3.95 kg, presented with no birth asphyxia and an unremarkable family medical history. Prenatal fetal ultrasound of the mother indicated bilateral ventricular enlargement, third ventricle enlargement, and posterior fossa cyst enlargement. Starting at 3 months of age, he began experienced seizures characterized by loss of consciousness, fixed gaze, cyanotic lips, frothy saliva, stiff limb tremors, and urinary incontinence. These seizures, lasting approximately 3 min, occurring multiple times a day. He was admitted to the pediatric intensive care unit, where the seizures persisted, characterized by fixed gaze, frothy saliva, limb tremors, lasting for more than 10 s, clinically suspected to be Dandy-Walker syndrome. Video electroencephalogram (EEG) showed no abnormalities, and no anticonvulsant medication was administered at that time. Following discharge, occasional seizures were reported, with no occurrences noted in the past year. His developmental progress significantly trails behind his peers. MRI scans showed reduced-sized bilateral cerebellar hemispheres, a visible midline cleft, underdeveloped cerebellar vermis, enlarged cisterna magna, mildly dilated lateral ventricles, a thin corpus callosum centered in the midline, and no significant bone or soft tissue abnormalities.

By the age of 1, the patient did not respond to his name, lacked eye contact, and although he could sit without support and roll over, he was unable to crawl and needed assistance to stand unsteadily. EEG recordings displayed the presence of numerous multifocal spike waves, spike slow waves, and slow wave discharges during wakefulness and sleep, along with widespread spike slow waves and multiple spike slow wave discharges. Subsequent head MRI scans showed an increase in the thickness of the ventricular system and narrowed subdural space under the frontal and temporal bones compared to previous findings. The brain parenchyma was unchanged from previous scans, with no high signal observed on the DWI sequence and the midline structure remained central without any notable abnormal signal foci in the skull and scalp soft tissues.

**Fig. 1:**
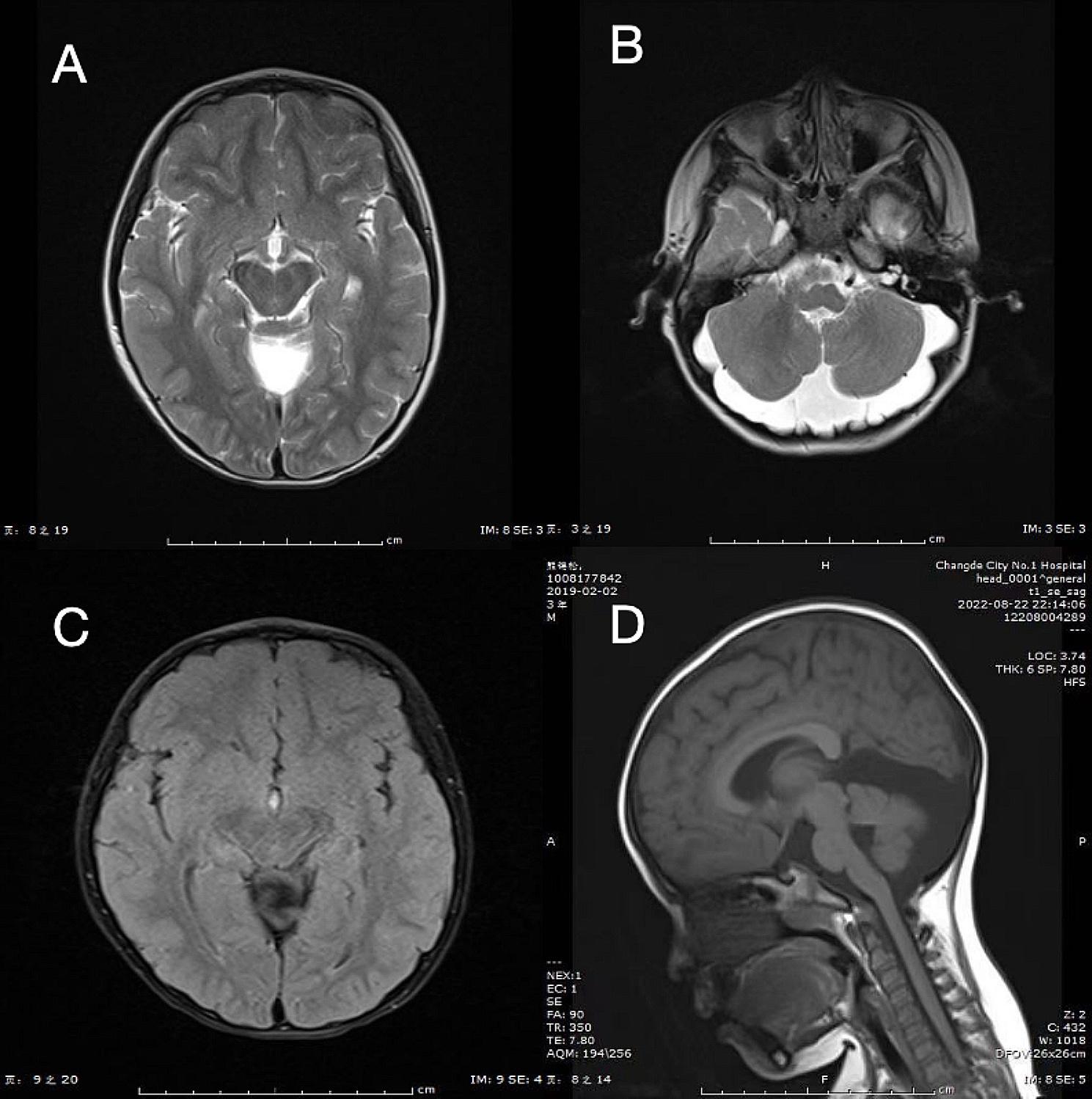
axi T2W1 **(A)**, axi T2W1 **(B)**, axi T2FLAIR v **(C)**, and sag T1W1 **(D)** magnetic resonance images of the proband. Note: Bilateral cerebellar hemispheres are small, cerebellar vermis is asymmetrical, cisterna occipitalis, cisterna quadrimata and ventricles are enlarged, bilateral ventricles are slightly widened, hydrazine bodies are thin, midline is centered, bone and soft tissue are not significantly abnormal. Diagnosis: cerebellar vermis changes, abnormal development? Hydrocephalus

After one year rehabilitation training began at the age of one, he had begun walking and speaking simple words such as ‘baba’ and ‘mama’. Currently, he is able to run, jump using both feet, and ascend stairs. Head MRI revealed smaller bilateral cerebellar hemispheres, an asymmetric vermis, an enlarged cisterna magna, a quadrigeminal cistern, and ventricles, with mildly dilated lateral ventricles, a thin corpus callosum, and a centered midline. No abnormalities were detected in the bone or soft tissues, as illustrated in Fig. 1.

### Trio-WES analysis

We employed a Trio-WES approach to identify causal variants within family trios. In brief, genomic DNA was extracted and enriched using a Qiagen DNA Blood Midi/Mini kit (Qiagen GmbH, Hilden, Germany) following standard protocols. Subsequently, 50 nanograms of DNA were fragmented to approximately 200 base pairs using fragmentation enzymes. The resulting DNA fragments were end-repaired and an A base was added to the 3’ end. Following this, the DNA fragments were ligated with barcoded sequencing adaptors, and fragments of approximately 320 base pairs were collected using XP beads.

After PCR amplification, the DNA fragments were hybridized and captured by NanoWES, in accordance with the manufacturer’s protocol. The hybrid products were eluted, collected, and subjected to further PCR amplification and purification. The libraries were then quantified using qPCR, and size distribution was determined using NanoWES (Berry Genomics, China). The genomic sequencing was conducted using the Novaseq6000 platform (Illumina, San Diego, USA) in 150 bp pair-end sequencing mode. Raw image files were processed using CASAVA v1.82 for base calling and generating raw data.

Alignment of the sequencing reads to the human reference genome (hg19/GRCh37) was performed using the Burrows–Wheeler Aligner tool, with PCR duplicates subsequently removed using Picard v1.57 (http://picard.sourceforge.net/). Variant calling was conducted using the Verita Trekker^®^ Variants Detection System by Berry Genomics and the third-party software GATK (https://software.broadinstitute.org/gatk/). Variant annotation and interpretation were carried out using ANNOVAR [14] and the Enliven^®^ Variants Annotation Interpretation System authorized by Berry Genomics. Annotation databases primarily included human population databases such as gnomAD, the 1000 Genome Project, Berrybig data population database, dbSNP, among others; in silico prediction algorithms including SIFT, FATHMM, MutationAssessor, CADD, SPIDEX; and disease and phenotype databases such as OMIM, ClinVar, HGMD, HPO.

Variants were categorized as ‘pathogenic,’ ‘likely pathogenic,’ ‘of uncertain significance,’ ‘likely benign,’ or ‘benign,’ in accordance with the American College of Medical Genetics and Genomics (ACMG) guidelines for interpreting genetic variants [15]. Variants with minor allele frequencies (MAF) < 1% in the exonic region or those with splicing impact underwent thorough interpretation, taking into account the ACMG category, evidence of pathogenicity, clinical synopsis, and inheritance model of the associated disease.For trio-analysis, potential monogenetic inheritance patterns, including de novo, autosomal recessive, autosomal dominant, X-linked recessive inheritance, mitochondrial, and, where possible, imprinted gene variation, were analysed. Variants found in the parents or recorded in any of the above-mentioned databases or in our in-house control exomes were excluded as the etiology.The Integrated Genomics Viewer was utilized to assess variants considered to be the cause of a recessive disorder. Manual inspection was conducted to evaluate coverage and identify additional variants across the entire coding domain [14–16].

### Minigene splicing assays

We designed two sets of nested primers, namely 230,300-F and 232,745-R, and 230,567-F and 232,446-R, for the nested PCR using normal human DNA as template. The wild-type and mutant minigenes were constructed into the pcMINI and pcMINI-C vectors. A partial Intron10 (480 bp) - Exon11 (92 bp) - partial Intron11 (562 bp) was inserted into the pcMINI vector, which contains a ExonA-IntronA-MCS-IntronB-ExonB structure. Additionally, a partial Intron10 (270 bp) - Exon11 (92 bp) - partial Intron11 (1066 bp) - Exon12 (79 bp) was inserted into the pcMINI-C vector featuring an ExonA-IntronA-MCS structure. After subjecting the samples to a 2-hour digestion reaction at 37 °C, we validated the results through electrophoresis and isolated the specific bands from the gel. After overnight ligation at 4 °C, the vectors were transformed into DH5α competent cells for an overnight incubation at 37 °C. Next, the transformed vectors were identified in colony/liquid culture PCR and Sanger sequencing.

Following the Lipofectamine™ 2000 protocol, four recombinant vectors were transfected into HeLa and 293T cells. Cells were pre-cultured to reach 70–80% confluence. On the day of transfection, vectors were prepared and diluted. Each transfection involved mixing 1 µg of plasmid DNA with 50 µL of serum-free Opti-MEM^®^ I Medium and 2 µL of Lipofectamine™ 2000. After a 5-minute incubation, the diluted Lipofectamine™ 2000 was added to the DNA solution, followed by a 20-minute incubation to form DNA-Lipid complexes. These complexes were then added to the cells and incubated for 4–6 h. The medium was replaced to avoid cytotoxic effects of the reagent. Cells were harvested for RNA extraction 48 h post-transfection.

For RNA extraction and reverse transcription, we utilized the RNeasy Mini Kit (Qiagen, Germany) following the manufacturer’s protocol. The process began with the disruption and homogenization of our samples. The lysate obtained was then run through a silica membrane for RNA binding, followed by a series of washing steps to remove impurities. To elute the RNA, we added RNase-free water directly to the silica membrane. Subsequently, the extracted RNA was subjected to reverse transcription using the High-Capacity cDNA Reverse Transcription Kit (Applied Biosystems, USA). Both the extracted RNA and the master mix (containing dNTPs, reverse transcriptase, buffer, and the primers) were combined in a PCR tube and subjected to the thermal cycling conditions specified by the kit protocol.

We utilized pcMINI-F/pcMINI-R primers (sequences provided in Table 1) for the amplification of pcMINI-wt/mut, and utilized pcMINI-C-F/pcMINI-C-R primers (sequences provided in Table 1) for pcMINI-C-wt/mut. The amplicons were determined with agarose gel electrophoresis and subsequently isolated for subsequent Sanger sequencing.

**Table 1:** Primers

Primer	Primer sequence(5′-3′)
230,300-F	agttcgagaccaccctgacc
230,567-F	ctgtatcacatggtctctgt
232,446-R	acctcaattgggatcatcca
232,745-R	tgggttctagtgtcgaaaat
235,180-F	gtcacctagtaagcattcag
236,089-R	agggagctgagagatctgca
pcMINI-OPHN1-KpnI-F	ggtaGGTACCgaaatgtttttctccctcct
OPHN1-mut-F	AGAAACTAATGAAAGAtaagctgtgccgctg
OPHN1-mut-R	cagcggcacagcttaTCTTTCATTAGTTTCT
pcMINI-OPHN1-XhoI-R	tttcCTCGAGagtccaaaatgtgcaagcct
OPHN1-overlap-F	tatctcaaccttttattcaagcacaatggg
OPHN1-overlap-R	cccattgtgcttgaataaaaggttgagata
pcMINI-C-OPHN1-KpnI-F	ggtaGGTACCtcctattaagtcttccgtct
pcMINI-C-OPHN1-XhoI-R	TAGACTCGAGAGGTTCTTTCCCATCCATGG
pcMINI-F	ACTTAAGCTTatgagtgggctttggggtggccggtt
pcMINI-R	TAGAAGGCACAGTCGAGG
pcMINI-C-F	ACTTAAGCTTatgagtgggctttggggtggccggtt
pcMINI-C-R	TAGAAGGCACAGTCGAGG

## Results

### Gene analysis results

**Fig. 2:**
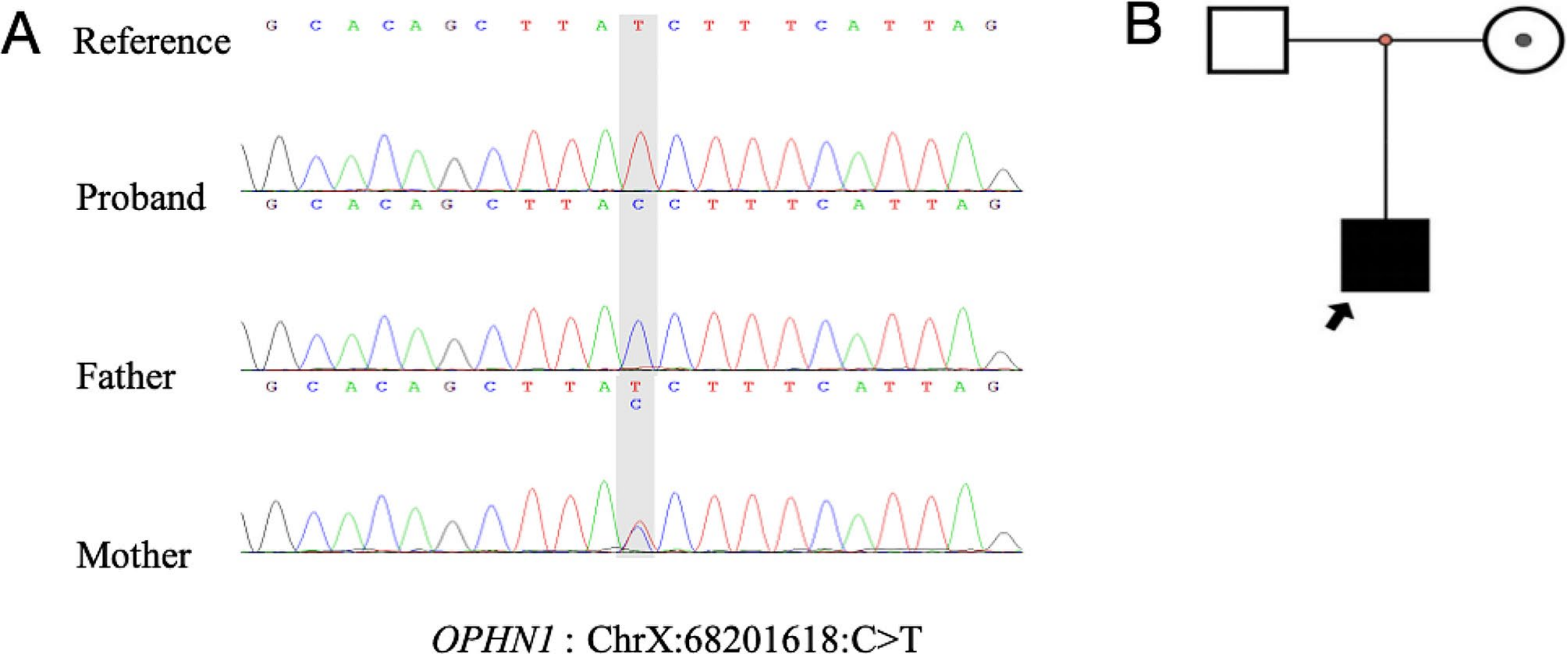
**(A)** Sanger sequencing of the *OPHN1*: c.1025 + 1G > A variant of family; **(B)** Family tree of this study

The WES revealed a hemizygous mutation in the *OPHN1* gene at the chromosome location ChrX: 68,201,618, specifically at NM_002547.3:exon11: c.1025 + 1G > A. The analysis for the family indicated that the father carries the wild type gene, while the mother also carries the gene variant. The ACMG classification for this gene variant was determined as “Likely Pathogenic”, presenting with PVS1 as a canonical splice site variant and PM2 as a variant which is not found in the occurrence frequency databases such as ExAC, gnomAD, and the 1000 Genomes Project East Asian population. The disease associated with this gene is “Mental retardation, X-linked, with cerebellar hypoplasia and distinctive facial appearance” [17], with phenotypic manifestations in OMIM including macrocephaly, prominent forehead, hypertelorism, long face, short philtrum, upturned nasal tip, periorbital wrinkles, prominent chin, large ears, narrow palpebral fissures, deep-set eyes, strabismus, nystagmus, long tubular nose, thin upper lip, cryptorchidism, micropenis, hypoplastic scrotum, and delayed psychomotor development, ranging from moderate to severe intellectual disability (IQ 40 to 60), or mild intellectual disability. This disease is characterized by an X-linked recessive inheritance, and the clinical presentation of the patient and the mother is consistent with this inheritance feature (shown in Fig. 2).

### Minigene splicing assays

**Fig. 3:**
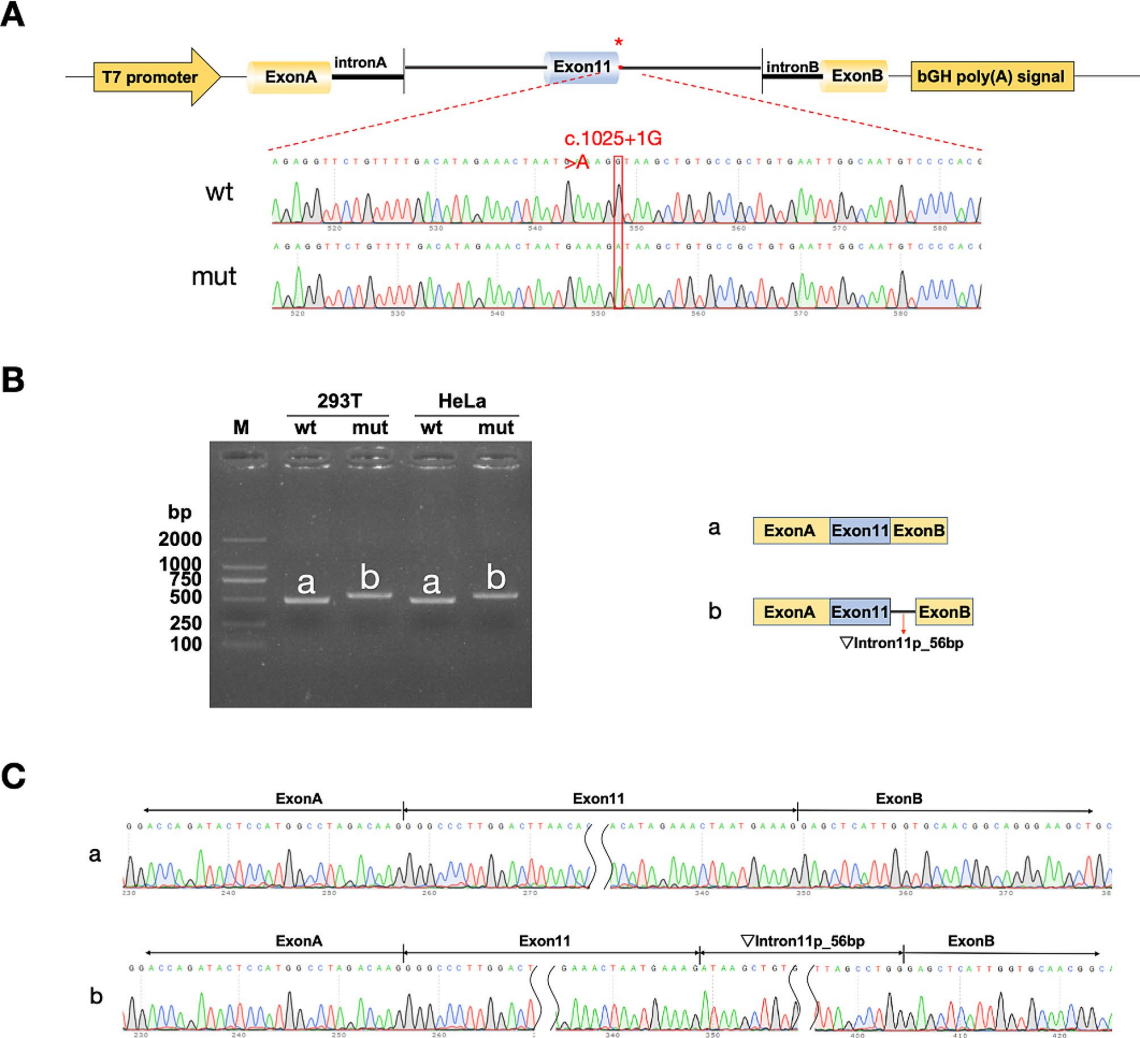
Results of pcMINI Vector Detection: **(A)** Schematic diagram and sequencing results of vector construction, with wt (wild type) displayed above and mut (mutation) below; **(B)** Gel electrophoresis and splicing diagrams of RT-PCR transcripts, with bands labeled as a and b in HeLa and 293T cells; **(C)** Sequencing results corresponding to the splicing bands. Red asterisks indicate the mutation site

**Fig. 4:**
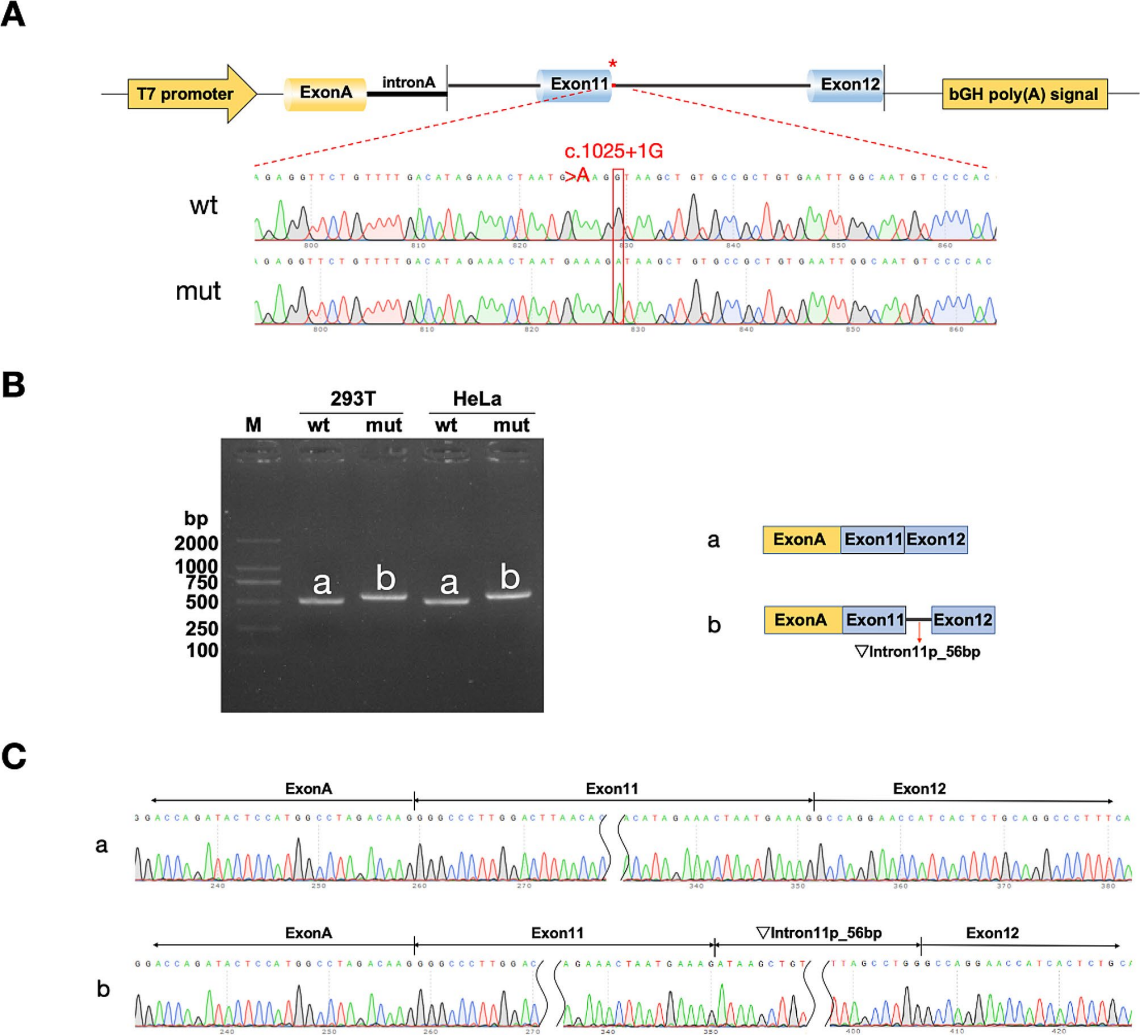
Results of pcMINI-C Vector Detection: **(A)** Schematic diagram and sequencing results of vector construction, with wt (wild type) displayed above and mut (mutation) below; **(B)** Gel electrophoresis and splicing diagrams of RT-PCR transcripts, with bands labeled as a and b in HeLa and 293T cells; **(C)** Sequencing results corresponding to the splicing bands. Red asterisks indicate the mutation site

The results of Sanger sequencing confirmed the successful integration of both wild-type and mutant minigenes into their respective vectors, as illustrated in Fig. 3 (Supplementary Fig. 1) and Fig. 4 (Supplementary Fig. 2). The in vitro minigene assays conducted in HeLa and 293T cell lines yielded consistent outcomes. Upon transfection with the pcMINI vector, both the wild type and the mutant manifested as single bands. The wild type conformed to the expected size (481 bp), whereas the mutant displayed an enlarged band. Sequencing of these bands revealed the wild type followed a conventional splicing pattern, consisting of ExonA (192 bp)-Exon11 (92 bp)-ExonB (57 bp). In contrast, the mutant showcased an irregular splicing sequence, incorporating a 56 bp segment on the left side of Intron11, displayed as ExonA (192 bp)-Exon11 (92 bp)-▽Intron11(56 bp)-ExonB (57 bp). In a similar vein, pcMINI-C vector transfection indicated that both cell types presented a single band for the wild type with a predicted size (509 bp), and an increased size for the mutant band. Sequencing indicated the wild type adhered to a regular splicing sequence of ExonA (192 bp)-Exon11 (92 bp)-Exon12 (79 bp), while the mutant illustrated an abnormal splicing pattern of ExonA (192 bp)-Exon11 (92 bp)-▽Intron11(56 bp)-Exon12(79 bp), with a retained 56 bp on the left side of Intron11.

The in vitro minigene experiments demonstrated that the c.1025 + 1G > A mutation impacts the splicing of gene mRNA. Both vector sets, pcMINI and pcMINI-C, produced analogous results, signifying the implications of the identified genetic variation. This genetic alteration led to a mutation at the translational level (c.1025_1026ins56bp, p.Pro343*), generating a premature termination codon (PTC) and resulting in a truncated protein comprising 342 amino acids.

## Discussion

In this study, we analyzed a Chinese Han family with XLID syndrome. We conducted Trio-WES to examine the genes associated with intellectual developmental disorders in the proband and their parents. We identified a novel variant c.1025 + 1 G > A in the coding region of the *OPHN1* gene, classified as a suspected pathogenic variant according to the ACMG classification guidelines. Subsequently, we used Sanger sequencing to validate this site in the proband and their parents, revealing that the proband’s mother carries this heterozygous mutation, reflecting the X-linked recessive inheritance of the disease. Importantly, this variant has not been previously reported, and there is no previous research evidence on the genetic variant and its impact on protein structure or function. Therefore, we constructed in vitro vectors containing the wild-type gene or gene variant and transfected these vectors into HeLa and 293T cell lines. Subsequently, we extracted total RNA from the cell samples and prepared cDNA for minigene construction. The investigation confirmed that the mutation c.1025 + 1G > A affects the splicing of the gene mRNA. The results from both sets of vectors, pcMINI and pcMINI-C, showed that the mutation leads to a 56 bp retention on the left side of Intron11. The 56 bp retention caused a PTC, resulting in a truncated protein with a length of 342 amino acids. Therefore, our demonstration confirms that the variant originating from the mother serves as the pathogenic cause of the patient, thereby contributing to the catalog of pathogenic sites within the *OPHN1* gene. The *OPHN1* gene encodes a Rho-GTPase-activating protein that facilitates the hydrolysis of GTP in Rho subfamily members. Rho proteins play a crucial role in intracellular signal transduction, influencing cell migration and morphogenesis. Variations in this gene have been implicated in OPHN1-related X-linked cognitive disability with cerebellar hypoplasia and distinctive facial dysmorphisms [18–20].

In an animal study, Khelfaoui et al. generated OPHN1-null mice and observed behavioral defects, including spatial memory impairment, social behavior deficits, lateralization issues, and hyperactivity. In a study on mouse cell culture, the inactivation of OPHN1 function led to an increase in the density and proportion of immature dendritic spines. The OPHN1 malfunction in the mouse model further confirmed the immaturity defect, demonstrating that OPHN1 is essential in all stages of development [21].

Numerous clinical studies have documented the association of the *OPHN1* gene with neurodevelopmental disorders. To date, over 100 cases of intellectual developmental delay linked to mutations in the *OPHN1* gene have been reported [22], encompassing more than 20 distinct mutation variants [23] known to contribute to conditions such as intellectual disability and autism(Supplementary Table 1). Billuart et al. [11] identified a 1-bp deletion in the *OPHN1* gene in a family case with X-linked syndromic intellectual developmental disorder. All four affected males carried the mutation, and seven unaffected females were carriers. Philip et al. [24] identified two different mutations in the *OPHN1* gene among affected members of two unrelated families with syndromic X-linked mental retardation. Zanni et al. [25] identified four different novel mutations in the *OPHN1* gene, with 2 (12%) out of 17 unrelated males with mental retardation and known cerebellar anomalies and in 2 (1%) out of 196 unrelated males with X-linked mental retardation without previous brain imaging studies.

From clinical reports, animal models, and protein functional studies, it is evident that the *OPHN1* gene is closely associated with the molecular mechanisms underlying the syndrome of XLID with cerebellar malformation and distinctive facial anomalies. Currently, the mutations recorded for *OPHN1* in the HGMD database are relatively limited, and the recorded mutation types mainly include missense, synonymous SNVs, frameshift substitutions, and stop gains. This case not only identifies the genetic factor for the patient’s condition but also adds to the catalog of pathogenic sites for *OPHN1* gene.

In conclusion, this study identified a novel mutation in the *OPHN1* gene associated with XLID. Through the analysis of the patient’s clinical manifestations and the mutated gene, it is hypothesized that the *OPHN1* gene variant is the cause of XLID in this particular case. For children presenting with seizures, intellectual disability, and visual impairment accompanied by cerebellar hypoplasia, it is recommended to include *OPHN1* gene mutation screening as part of the diagnostic process. Currently, our understanding of the molecular mechanisms underlying XLID remains incomplete. The findings of this study broaden the phenotypic and genetic spectrum of XLID, providing valuable insights for family counseling.

### Electronic supplementary material

Below is the link to the electronic supplementary material.

Supplementary Material 1. Supplementary Fig. 1. Original gel/blot imagesof pcMINI Vector Detection. Gel electrophoresis and splicing diagrams of RT-PCR transcripts, with bands labeled as a and b in HeLa and 293T cells. Capture photographs and obtain gel images utilizing the Tanon 1600 gel documentation system.

Supplementary Material 2. Supplementary Fig. 2. Original gel/blot imagesof pcMINI-C Vector Detection. Gel electrophoresis and splicing diagrams of RT-PCR transcripts, with bands labeled as a and b in HeLa and 293T cells. Capture photographs and obtain gel images utilizing the Tanon 1600 gel documentation system.

Supplementary Material 3

Supplementary Material 4

## Data Availability

The original contributions of this study are detailed within the article and its supplementary materials. The WES data related to this study have been deposited at the NCBI (https://www.ncbi.nlm.nih.gov) under the Project ID PRJNA1125921. For additional information, please contact the corresponding author.
