# Supplementary material for: Effect of the OPHN1 novel variant c.1025+1 G>A on RNA splicing: insights from a minigene assay
Source: BMC Med Genomics. 2024 Jul 2;17:175. doi: 10.1186/s12920-024-01952-1 (PMC11221095; doi:10.1186/s12920-024-01952-1)
Supplement: Supplementary file 2 — Supplementary Material 2. Supplementary Fig. 2. Original gel/blot imagesof pcMINI-C Vector Detection. Gel electrophoresis and splicing diagrams of RT-PCR transcripts, with bands labeled as a and b in HeLa and 293T cells. Capture photographs and obtain gel images utilizing the Tanon 1600 gel documentation system. [file 12920_2024_1952_MOESM2_ESM.docx]

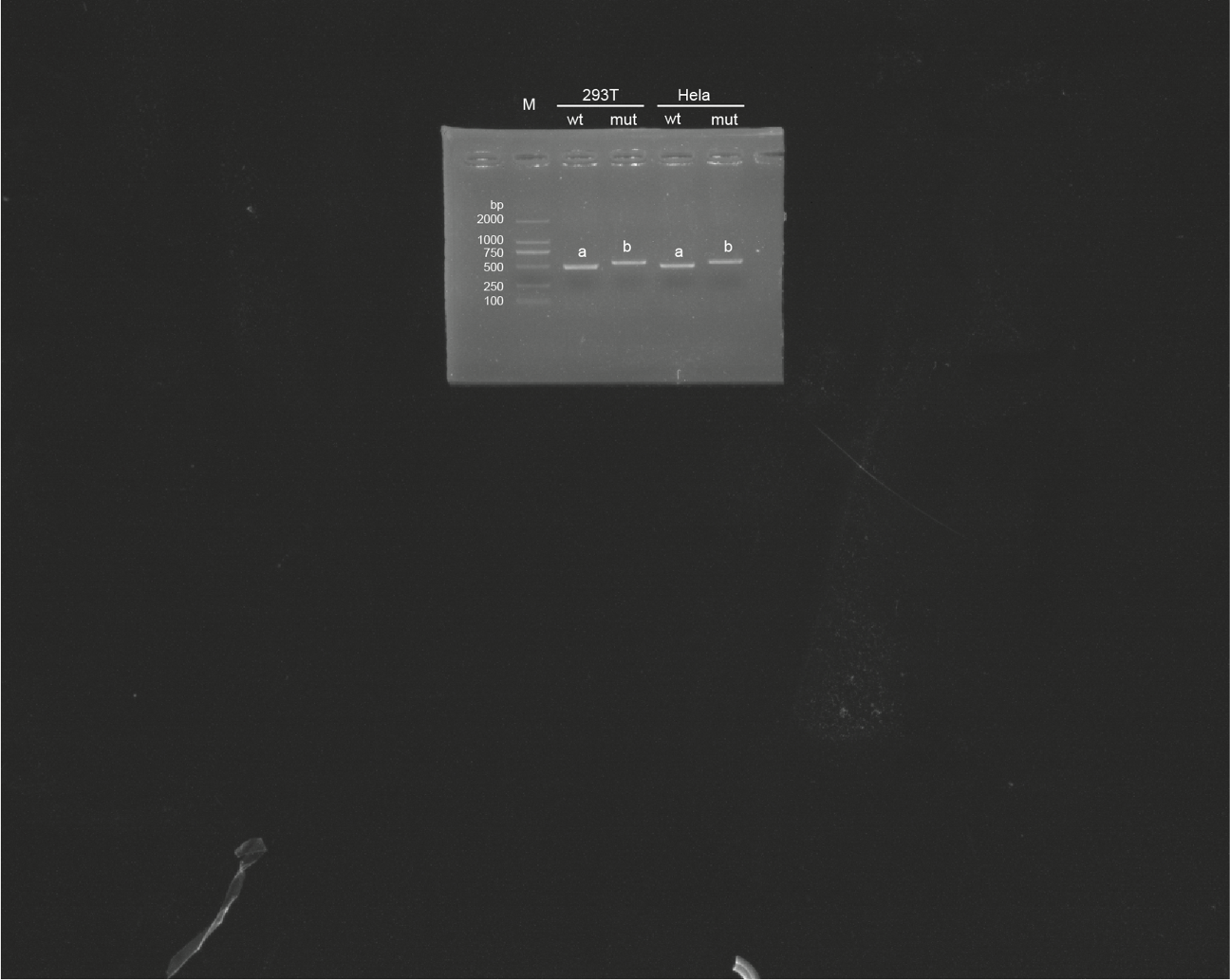


Supplementary Figure 1. Original gel/blot imagesof pcMINI Vector Detection. Gel electrophoresis and splicing diagrams of RT-PCR transcripts, with bands labeled as a and b in HeLa and 293T cells. Capture photographs and obtain gel images utilizing the Tanon 1600 gel documentation system.


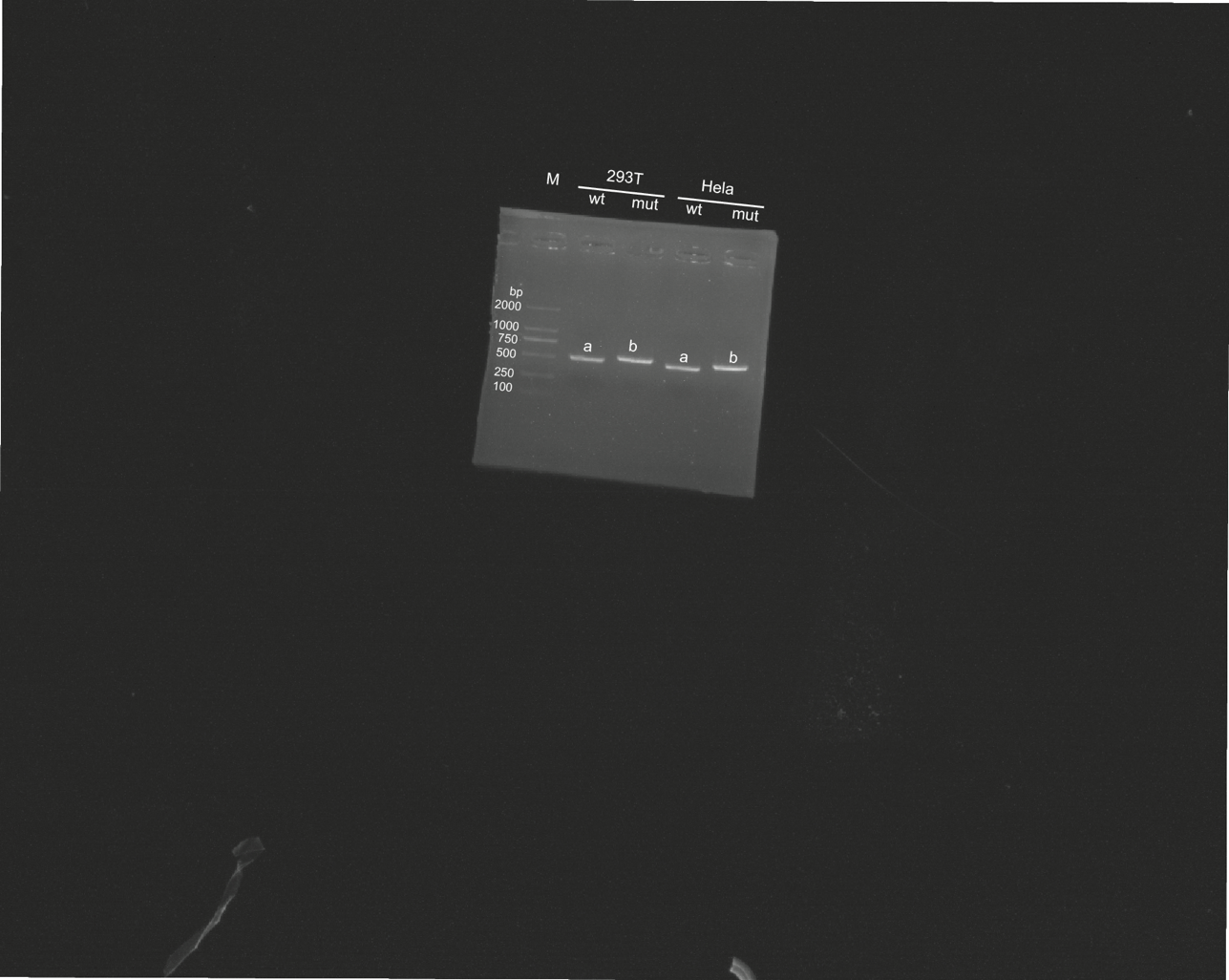


Supplementary Figure 2. Original gel/blot imagesof pcMINI-C Vector Detection. Gel electrophoresis and splicing diagrams of RT-PCR transcripts, with bands labeled as a and b in HeLa and 293T cells. Capture photographs and obtain gel images utilizing the Tanon 1600 gel documentation system.
