# Supplementary figures and images for: Effect of the OPHN1 novel variant c.1025+1 G>A on RNA splicing: insights from a minigene assay
Source: BMC Med Genomics. 2024 Jul 2;17:175. doi: 10.1186/s12920-024-01952-1 (PMC11221095; doi:10.1186/s12920-024-01952-1)

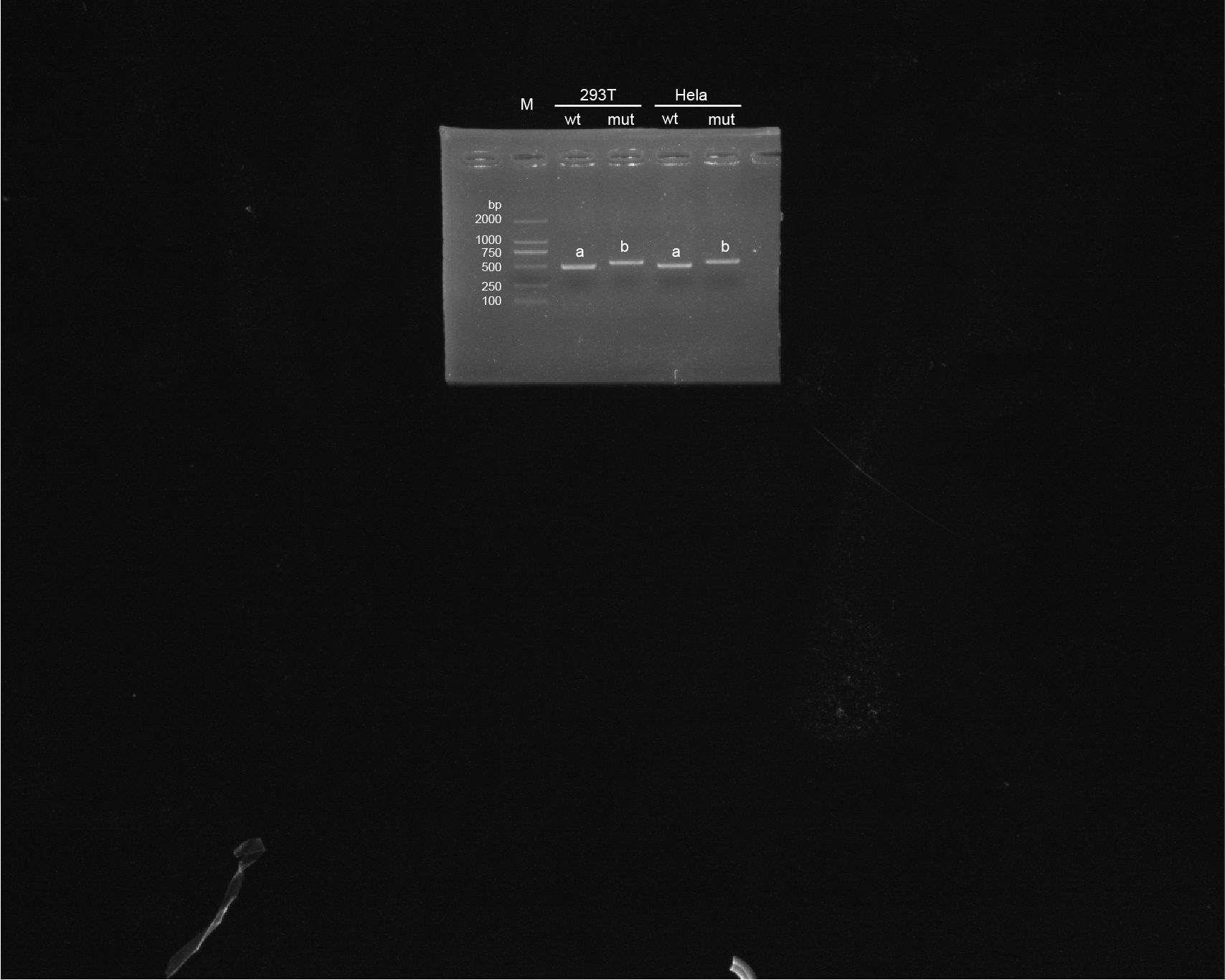

Supplement: Supplementary file 3 — Supplementary Material 3 [file 12920_2024_1952_MOESM3_ESM.tif]

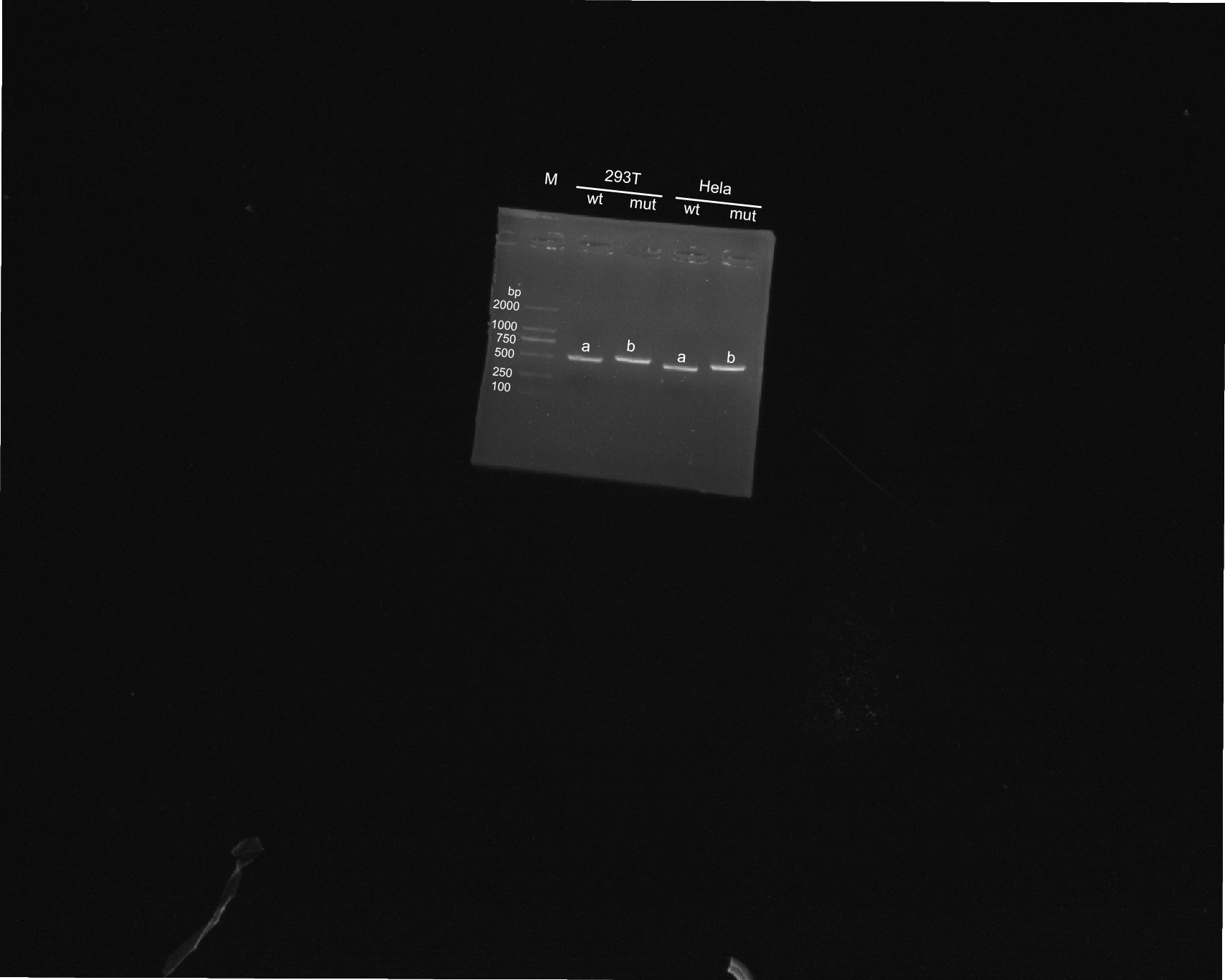

Supplement: Supplementary file 4 — Supplementary Material 4 [file 12920_2024_1952_MOESM4_ESM.tif]
